# Transactions of the Provincial Medical and Surgical Association

**Published:** 1845-10

**Authors:** 


### Art. XX.

*Transactions of the Provincial Medical and Surgical Association.* Vol. XIII.—*London,* 1845. 8vo, pp. 418.

We are glad to see that the council of the Association have changed their type. The volume will now contain nearly double the matter in the same space, and thus the cost of publication will in future be greatly lessened,—a consummation devoutly to be wished. The papers composing the present volume are unusually few in number, and some of them seem scarcely to come within the scope of Transactions published by a medical society. Indeed, we may say that, with the exception of the two excellent Anniversary Reports, the only thing in the volume,—strictly speaking,—that has a legitimate claim to be there, is the communication of Mr. Giles at the end, consisting literally of only two pages! The "Essay" of Mr. Crosse, however excellent in itself, as a "literary and practical" compendium of our knowledge respecting the subject of which it treats, has fewer pretensions to the character of originality than memoirs published as "Transactions" commonly possess. It is essentially *a book*, and ought decidedly to have been given to the world as such. It is much too good a monograph to be lost; and we doubt not its own merits and the high reputation of its author would have ensured for it a sale in the ordinary way of publication.

Dr. Hocken's elaborate essay on the Inflammatory affections of the Retina, seems to possess still fewer claims to the honour of the Association's *imprimatur*. It might have constituted a very tolerable chapter in his treatise of Diseases of the Eye, but how it came to find a place in the transactions of a Society, is not so obvious to our apprehension.

The volume, as usual, commences with the Retrospective Addresses delivered at the last anniversary meeting. These papers, from their nature, claim of right a place in the volume; and to this they are no less entitled by their intrinsic value.

I. *The Retrospective Address* [*on Practical Medicine.*] By Charles Cowan, m.d., Physician to the Royal Berkshire Hospital, Reading. The Report of Dr. Bennett, published in our last Number, comprehending as it does the whole of the period included in Dr. Cowan's address, is so full on the subject of practical medicine that we cannot be expected to do more than to advert to Dr. Cowan's production. It is, however, but justice to the author to state that it is extremely creditable to him, and will stand comparison with any of its predecessors. It is not and does not pretend to be so comprehensive or complete as Dr. Bennett's Report, but it embodies a vast number of valuable facts, well arranged, and clearly described. In one of its most interesting parts,—the biographical section,—the Address has the advantage over our Report, the subject of biography not being comprehended in the scheme adopted in our Journal. We are of opinion that the future orators of the Association would do well to cultivate and extend this department of their subject, even to the exclusion of more practical matter. In an oral address, the details of experience in the various branches of medicine, however well stated, prove wearisome to the hearer, and often cannot be retained in the memory; while the interest attaching to the personal history and character of those who have trod the same path with ourselves, and have reached the goal we are all approaching, is deep and universal. Dr. Cowan's sketches are excellent as far as they go.

II. *Retrospect of Anatomy and Physiology for the year* 1843-44. By William Budd, m.d., Physician to St. Peter's Hospital, Bristol.

Of the subjects of Dr. William Budd's Report, a large proportion have been already noticed, under some form or other, in our own pages; and we shall therefore confine ourselves to a brief statement of its contents. After some general preliminary remarks, Dr. Budd proceeds at once to an enumeration of the principal discoveries in Organic Chemistry made during the preceding year; to which nearly half his Address is devoted. The principal experimenters whose results are introduced, are, of course, Liebig, Dumas, and Mulder; but a full account is also given of the researches of Blondlot, on the chemistry of digestion; of Dr. Percy on the elimination of sugar through the kidney; of Schwann and Bouisson on the bile; together with a notice of Scharling's experiments on respiration. He then takes up Chossat's experimental inquiries on inanition; to the results of which his evident *penchant* for chemical analysis prevents him, we think, from doing full justice. He thinks that nothing has been ascertained by M. Chossat, which had not been clearly laid down in principle, and almost in so many words, in various chemical works, especially those of Dumas and Liebig; but we doubt if any one, however fully acquainted with the *theory,* would have anticipated the *fact,* that a bird dying of starvation would be completely resuscitated by a sufficient amount of heat. We cannot quite accord with Dr. Budd's opinion of the equal or even superior value of chemistry to anatomy, in the education of the physiologist; and we are rather disposed to think that he is one who expects too much from the former science. But we would in nowise seek to hold back any who are inclined to devote themselves to its extension; being satisfied that its real value in medicine will be ultimately far greater than that which we can at present or in immediate prospect attribute to it.

The summary of anatomical inquiries (which we should have expected to see holding the foremost rank) next follows; and here we meet with numerous names, among which those of Foville, Stilling, Bidder, Volkmann, and Newport are the chief. The valuable paper of the last-named author is not noticed by any means as fully as it appears to us to deserve,—its bearing on general questions of physiology being taken into account. A full analysis is given of Matteuci's experiments on animal electricity; and this derives an increased value from the interesting suggestions founded by Dr. Budd upon these researches, in regard to the mutual relations of electricity and nervous power. A notice of the complete identification, by Bischoff, Raciborski, and others, of the menstrual period in the human female with the rut in other animals, is the chief remaining feature in the address. We have only to say, in regard to its composition and execution, that, like Dr. Budd's former productions, it evinces not only industry in collecting facts, but that sagacity in valuing them, which is the characteristic of a philosophic mind; whilst the diction and style indicate the author's high literary cultivation. Well will it be for the Association, if its future Retrospective Addresses in this department, always come up to the standard which Dr. Budd has thus set.

III. *The Varieties, Causes, Pathology, and Treatment of the Inflammatory Affections of the Retina.* By E. O. Hocken, M.D. Deep-seated, internal inflammation of the eyeball, although it very often implicates both choroid and retina, may have its seat, sometimes more especially in the choroid (*choroiditis*),—sometimes more especially in the retina (*retinitis*). But authors appear to differ as to which case should be called choroiditis and which retinitis;—by some the choroid is considered the seat of the disease when the principal symptom is merely dimness of vision;—the retina, when to dimness of vision there is conjoined fiery spectra and intolerance of light;—by others, on the contrary, and we are disposed to agree with them, dimness of vision alone is viewed as the characteristic of retinitis; and fiery spectra and intolerance of light that of choroiditis.

This uncertainty depends on the absence, at first, of objective symptoms,—the sclerotic vascular injection around the cornea and other appearances being symptomatic merely of the anterior internal inflammation, iritis, &c.,—which almost always coexists, in a greater or less degree,—not of the deeper-seated inflammation.

The deep-seated internal inflammation of the eyeball to which Dr. Hocken refers in the paper before us, is that which is especially characterized by the symptoms of fiery spectra and intolerance of light. He commences at once to speak of it as retinitis without even defining what retinitis is—without inquiring what objective and subjective phenomena are peculiar to it, and what objective and subjective phenomena, which, although usually coexisting with it, are not peculiar to it, but to attendant inflammation of other parts of the eye.

Although Dr. H. speaks of changes which the retina undergoes from loss of transparency, effusion of lymph, displacement, &c., and which he says may be readily seen and appreciated during life by the practised and careful observer, almost as well as if exposed to the scalpel, he does not describe in detail these changes, as he might have done, if he had so readily seen and appreciated them, nor does he show their connexion with the subjective symptoms of the inflammation: and yet these are the very things which we want to know, and which alone could have given Dr. Hocken's paper any pretensions to novelty and interest, and entitled it to occupy so large a space in the Transactions of any Scientific Association.

IV. *An Essay, literary and practical, on Inversio Uteri.* By J. G. Crosse, Senior Surgeon to the Norfolk and Norwich Hospital. This paper is incomplete; the causes, diagnosis, and treatment of inversio uteri being reserved as the subject of a second communication. When completed, we shall not fail to notice the whole essay. The part before us contains the facts on which,—as he informs us at p. 344,—he will, in his next paper, proceed to reason. The most valuable portion of the paper now published are the account of the chronic inversion of the uterus, and the history of those cases in which the unimpregnated uterus has been inverted by tumours, &c. The part of the essay which treats of inversion occurring during labour, or immediately afterwards, is less satisfactory; since the period of its occurrence, the frequency of hemorrhage, &c., and some other points connected with it,—which much need elucidation by a careful collation of the numerous cases on record,—are not noticed. We presume, however, from what the author says at p. 309, that he will enter fully into these inquiries in his next part. We have no doubt that this memoir, when finished, will be the most complete monograph on the subject in any language, and will amply sustain the author's high reputation for learning and professional skill.

V. *Case of Congenital Malformation of the Urinary Organs.* By Henry Giles, Esq. As this is an interesting example of a curious class of cases, we give it entire. It is illustrated by a plate.

"Francis Hyde is now about 22 years of age, in good health, and capable of considerable exertion; he is small, but not ill-formed, with the exception of the parts to be noticed. A tumour presented itself at the lower part of the abdomen, of a bright red appearance; this on examination is found to be the bladder, presenting its mucous surface without any covering or protection. It passes from its proper cavity beneath the symphysis pubis, and between the cura of the corpora cavernosa of the penis, previous to their union; after passing under the pubis it turns upwards and rather backwards, overlapping the symphysis and pressing against the centre of the lower part of the abdomen, which has become concave for its reception, giving the idea, at first sight, that it passed out above the symphysis. The protruded portion is generally about the size represented in the drawing [a walnut]; sometimes it is larger, at others smaller, contracting so much that it could be covered with a shilling, and appearing as though it were likely to be completely withdrawn. I formed an idea that this state was produced voluntarily, but found him incapable of exercising the least control over it. Externally and inferiorly, on both sides, the ureters open on the exposed surface, from which the urine is seen oozing, generally in drops; but if a little liquid be given him, it is thrown out in a small jet; this is also the case on his rising after lying down for a few minutes; very little forms during the night; it is at all times exceedingly acrid, and deposits large quantities of sand. Immediately under the protruded bladder, and to some extent supporting it, are the rudiments of a penis; it seems to be formed chiefly of the corpora cavernosa, united along their inferior and internal margins; superiorly they are turned outwards, and form a flat surface, on which, as before stated, the bladder rests: this is covered with a sort of fibrous membrane, which presents several openings of various sizes. There are no traces of either an urethra or glans penis. At the union of the corpora cavernosa anteriorly is placed a species of frenum. The scrotum is well formed, and the testicles are fully developed. He occasionally experiences strong venereal excitement; the penis at these times becomes somewhat distended and elongated; the seminal fluid oozes between it and the bladder. From constant efforts to protect the parts in walking, the inferior extremities have become bent outwards at the knee, making his gait extremely awkward." (pp. 351-2.)

---

